# A New Tool for Quantifying Mouse Facial Expressions

**DOI:** 10.1523/ENEURO.0349-22.2022

**Published:** 2023-02-22

**Authors:** Olivia Le Moëne, Max Larsson

**Affiliations:** Division of Neurobiology, Department of Biomedical and Clinical Sciences, Linköping University, Linköping 581 83, Sweden

**Keywords:** mouse grimace scale, mouse profile, optogenetics, positive emotion, somatosensation

## Abstract

Facial expressions are an increasingly used tool to assess emotional experience and affective state during experimental procedures in animal models. Previous studies have successfully related specific facial features with different positive and negative valence situations, most notably in relation to pain. However, characterizing and interpreting such expressions remains a major challenge. We identified seven easily visualizable facial parameters on mouse profiles, accounting for changes in eye, ear, mouth, snout and face orientation. We monitored their relative position on the face across time and throughout sequences of positive and aversive gustatory and somatosensory stimuli in freely moving mice. Facial parameters successfully captured response profiles to each stimulus and reflected spontaneous movements in response to stimulus valence, as well as contextual elements such as habituation. Notably, eye opening was increased by palatable tastants and innocuous touch, while this parameter was reduced by tasting a bitter solution and by painful stimuli. Mouse ear posture appears to convey a large part of emotional information. Facial expressions accurately depicted welfare and affective state in a time-sensitive manner, successfully correlating time-dependent stimulation. This study is the first to delineate rodent facial expression features in multiple positive valence situations, including in relation to affective touch. We suggest using this facial expression assay might provide mechanistic insights into emotional expression and improve the translational value of experimental studies in rodents on pain and other states.

## Significance Statement

The existence of distinct mouse facial expressions in painful situations is well established. However, the current methods in use do not offer features both visualizable by a human observer and finely quantifiable. To address this issue, we established seven new facial parameters, directly measurable on mouse profiles, that give insight into the facial regions responsible for changes in expression. This method is completely transparent and can be applied to a wide range of mammals. To complete the spectrum of known facial expressions, we submitted the mice to sequences of pleasant and aversive gustatory and somatosensory stimuli. Broadening the spectrum of experimental contexts amenable to facial expression analysis, including those of positive valence, can improve the translatability of animal emotion studies.

## Introduction

Emotions are defined as transient states, experienced and expressed through a multicomponent system, comprising behavioral and physiological responses, as well as a cognitive and a subjective component (Paul et al., 2005; Shuman et al., 2017). Although emotions are relatively easily identified in humans through verbal reports, their existence and identification in nonhuman animals have been debated for many years (de Vere and Kuczaj, 2016). In particular, the subjective component of any emotional experience is difficult to assess in animals, the verbal production of which remains largely unintelligible for researchers. Nonetheless, observations of neurophysiological changes accompanying exposure to pleasant and aversive stimuli, together with the inclusion of patterns of vocalizations, behavioral and facial displays point toward the existence of a wide range of emotional experiences in laboratory rodents, farm animals and various primates (Désiré et al., 2002; Morris et al., 2008; Makowska and Weary, 2013; Dolensek et al., 2020).

Emotions motivate a large aspect of our behaviors (Berridge, 2018) but can be difficult to recreate in laboratory conditions. The challenge of inducing and identifying them in animal models could contribute to the translational crisis experienced in neuroscience, with a large proportion of physiological, pharmacological or behavioral findings failing to be replicated from the lab to the clinic (Drucker, 2016; Fendt et al., 2020). Most mammals, including humans, can produce facial movements which Darwin had already postulated to be highly conserved between species (Darwin, 1872). Such movements, and their capture in facial expressions are considered the richest source of emotional information (Ekman, 2009). Facial expressions give insight into spontaneous, momentary affect in response to a stimulus or to the memories of its consequences, motivating a physiological and behavioral reaction (Webb et al., 2019). Understanding and interpreting animal emotions is especially relevant to understand social species who have a mutual benefit in communicating and interpreting each other’s emotional state.

The potential of facial expressions for emotion decoding was already systematically assessed earlier by (Grill and Norgren, 1978), and they have since successfully been used to assess pain, pleasure, malaise or bitter tasting in several species (Langford et al., 2010; Defensor et al., 2012; Dolensek et al., 2020). Most facial expressions analyses in rodents are based on the pioneer work of Langford and colleagues (Langford et al., 2010) and the development of their Mouse Grimace Scale (MGS). This scale, and those derived, usually focus on three major facial areas: eyes, ears and muzzle; and rely on a three-point rating of facial expressions of pain: 0 “absent,” 1 “moderate” or “inconclusive,” 2 “obvious.” Despite the successful establishment of the MGS, and the subsequent rising interest for animal facial expressions, most of the research in the field focuses on pain responses, and little work has been done on other negative affective scales (e.g., fear, frustration, anxiety) or positive ones (Mogil et al., 2020).

Positive emotions are notoriously difficult to observe or quantify in animals, compared with negative affective states (Paul et al., 2005). An earlier study found no changes in MGS scores in a positive situation elicited by rat tickling, suggesting that these grimaces might be almost exclusively related to pain (Finlayson et al., 2016). Sucrose tasting, or foods conveying an equivalent hedonic value are the gold standard to evaluate positive affective states. However, this solution is not free of confounds, as tasting per se requires the solicitation of facial muscles, and food reward alone cannot accurately represent the entire range of positive affects. Moreover, defining boundaries between different emotions is challenging (Ekman, 2009), and complex emotions can overlap. Consequently, affective states are regarded as a continuum rather than categorical entities. With regards to these concerns, research still lacks quantitative methods to identify and quantify affective states in animals (Middleton et al., 2021). MGS-based assessments lack fine quantification of facial changes, and newly developed methods using machine-learning algorithms, although powerful, do not provide an understanding of the processes underpinning facial expressions categorization and interpretation. Moreover, the latter may not be easily adaptable to freely behaving animals.

In this study, we propose a new facial expression model based on features both visualizable by a human observer and finely quantifiable for analysis. We compare these features across a range of different stimuli, including two pain models as well as positive and aversive tastants. To extend the range of positive stimuli tested on facial expressions, we include several non-noxious somatosensory stimuli. Gentle, affiliative touch signaled through low-threshold C-fiber mechanoreceptors (C-LTMRs) carries a positive affective value in a wide range of species including social rodents (Cho et al., 2021; Pawling et al., 2017). We analyzed the changes in facial expression over sequences of emotion-inducing stimuli to (1) establish the facial expression associated to each stimulation, (2) determine whether different stimulations differentially affect facial features, and (3) identify the facial features conveying the most emotional valence. Finally, while facial expressions in theory convey emotions in a time-sensitive manner, few studies have exploited the dynamics of expressions changes. Therefore, we monitored facial expressions surrounding brief optogenetic stimulation of CGRP expressing nociceptors. Similarly, we analyzed facial changes over time in a well-established pain assay using formalin, a compound known to elicit two phases of pain behavior separated by an inactive phase, hypothesizing that facial expressions would show a similar pattern.

## Materials and Methods

### Animals

Five experiments were conducted, including a total of 90 C57BL/6JRj mice from Janvier Laboratories and 16 CGRP;LSL-ReaChR mice bred in house (see details below, Optogenetic stimulation; 62 females (F): 10.8 ± 0.2 weeks old, 20.5 ± 0.2 g; and 44 males (M), 10.5 ± 0.2 weeks old, 26.7 ± 0.4 g on testing). The mice were housed in NexGen IVC system Mouse 500 at 20 ± 2°C, under a 12/12 h light/dark cycle (12L:12D light cycle), lights being on at 7 A.M. Mice included in the first experiment [sucrose-mentholatum-petting (SMP); *n* = 45] were previously used in an unrelated study without experimental intervention, for 6 d before the experiment, where they were submitted to the same temperature and light cycle. In this previous experiment, groups of five individuals (4 F + 1 M) were housed together in an enriched semi-natural environment. For identification purpose, some of these mice had different patterns shaved on the neck or the back.

All experimental procedures employed in the present experiment were approved by the Local Ethics Committee for Animal Experiments at Linköping University; and in agreement with the European Union council directive 2010/63/EU.

### Apparatus

The experimental box was a clear Plexiglas cubicle of dimensions 9 cm long × 5 cm high × 5 cm wide, pierced with holes on the roof to allow for ventilation, and on the side to introduce a pipet containing tastants. The box was cleaned and disinfected between each individual session.

### Procedure

Mice were individually introduced to the experimental cubicle where their facial expressions were recorded from the side, using a monochrome Basler ace camera (acA1300-60gm) or a color FLIR camera (Blackfly BFS-U3-23S3C-C) with a 1:1.8/4 mm Basler lens (C125-0418-5M) and the Open Broadcaster Software 27.0.1 (https://obsproject.com/). All experiments took place between 9 A.M. and 13 P.M., during the light phase of the light cycle. Following experiment completion, animals were killed using carbon dioxide asphyxiation and cervical dislocation.

#### Experiment SMP

Forty-five mice (36 F, 9 M) were included in the SMP experiment consisting of a sucrose-mentholatum-petting (SMP) stimulus sequence in a fixed order, for 5 min each. Each mouse was successively placed in the cubicle, where it was left undisturbed for 5 min to establish a baseline, and then submitted to the sequence. The first stimulus was a 20% sucrose solution (84100, Sigma-Aldrich) introduced by a pipet through the hole pierced in the short side of the cubicle. Tasting was entirely voluntary from the mice. Following sucrose stimulus, a second pipet was introduced containing a bitter solution. This solution was prepared by diluting three drops of anti-bite nail polish (Mentholatum) into 10 ml of water. Finally, the mice were gently petted by the experimenter’s finger, from the base of the neck to the lower back, at a velocity averaging 5 cm/s. Among the 45 mice included in this study, 20 (16 F, 4 M) were naive to petting, and 25 had been habituated to it. The habituation protocol consisted in petting the mice 5 min every day, for 6 d before the experiment day. The stimuli-sequence followed a fixed order to optimize taste differentiation, avoid solution contamination and maximize the accuracy of the elicited emotional state. The bitter stimulus, mentholatum, was given after sucrose as aversive stimuli produce stronger responses than palatable ones. In addition, bitterness tends to be a persistent taste that could have confounded sucrose results. Petting the mice required opening the cubicle, which could cause the mouse to escape, thus it was kept as the last stimulus.

#### Experiment SQB

Twenty mice (10 F, 10 M) were included in experiment SQB, which consisted of the stimulus sequence sucrose-quinine-brushing in a fixed order, for 5 min each. The same procedure as in experiment SMP was applied. The quinine stimulus was a 0.8 m quinine solution (22630, Sigma-Aldrich). This concentration produced a distinctively less bitter taste than the mentholatum dilution. Brushing was performed by the experimenter with a small paint brush made of synthetic hair (Panduro, 1-cm-wide flat tip), using the same pressure and velocity as in the petting phase of the SMP experiment. Ten mice (5 F, 5 M) were habituated to brushing before the experiment. Habituated mice were individually brushed for 5 min each, every 2 d, for 12 d before the SQB experiment, resulting in six habituation sessions.

#### Cutaneous touch experiment

Twenty mice (10 F, 10 M) were individually placed into the observation box, where after a 5-min baseline, they were given water to taste through a pipet. Notably, the mice were not water-deprived before experiment. Then, the mice were submitted to a sequence of three cutaneous stimuli in a randomized order. The mice were petted by the experimenter’s finger, as in experiment SMP, brushed with the small paint brush as in experiment SQB, and gently poked. Poking consisted of applying the tip of the brush handle on the neck of the mouse, with the same pressure as during brushing and petting, but without motion. Half of the mice had been habituated to the cutaneous stimuli before the experiment, following the same habituation protocol as in experiment SQB.

#### Formalin test

Twenty mice (10 F, 10 M) went through the formalin test, among which six males and nine females were reused from the SQB experiment. In the first phase of the experiment, the mice were introduced to the observation cubicle, and left undisturbed for 30 min to establish a baseline. Immediately after the end of baseline, each mouse was placed back in its home cage for 5 min to allow for drinking and feeding. Afterwards, the mouse was quickly brought to an anesthesia chamber where it was lightly anesthetized with isoflurane (3.5%). The mouse then received a single 20-μl intradermal injection of either vehicle (saline, 9 mg/ml, *n* = 10, 5 F, 5 M), or formalin (2% in saline, from Sigma-Aldrich F8775) in the plantar hind paw. When the injection was completed, the mouse was brought back into the cubicle. The observation resumed as soon as the mouse woke up, and carried on for the next 30 min. The interval between the beginning of the anesthesia and the beginning of the second observation phase was <5 min.

#### Optogenetic stimulation

In order to optogenetically target a broad population of primary afferent nociceptors, we used a recombinase dependent mouse line, Calca^Cre^;R26^LSL_ReaChR^ which expressed the red-shifted channelrhodopsin ReaChR (Lin et al., 2013) under the control of Cre expressed from the Calca [encoding calcitonin gene-related peptide (CGRP)] locus (referred to as Het mice in the rest of the manuscript). These mice were generated by crossing homozygous Calca^Cre^ mice (#033168, The Jackson Laboratory; Carter et al., 2013) with heterozygous R26^LSL_ReaChR^ mice. The latter mice were in turn generated by crossing R26^LSL_FSF_ReaChR-mCitrine^ mice (#024846, The Jackson Laboratory) with a FlpO deleter mouse strain, B6 ROSA26Flpo (#012930, The Jackson Laboratory; Raymond and Soriano, 2007). As control, we used littermate LSL_ReaChR negative mice (referred to as WT).

Sixteen (5 F, 11 M) mice were included in the experiment, including nine Het (2 F, 7 M) and seven WT controls (3 F, 4 M). The mice were placed in the observation box and left to habituate for 3 min. Optogenetic stimulation was powered by a LED current driver through a 595-nm LED (Doric Lenses). Light was delivered to the base of the neck with an optical fiber patch cord (length 1 m; 0.63 NA; 960 μm in diameter) producing 19.2-mW power at the tip of the fiber; the tip was placed an estimated average distance of 3 mm from the surface of the skin as measured from the video recordings, covering ∼25 mm^2^ of the skin surface. The mice were stimulated for 3 min at 20 Hz with 10-ms pulses. Following the end of optogenetic stimulation, the mice were left to recover for 3 min. All mice were shaved 24 h before the experiment on the stimulation area at the base of the neck to ensure direct light exposure to the skin.

### Facial parameters

**Figure 1.**
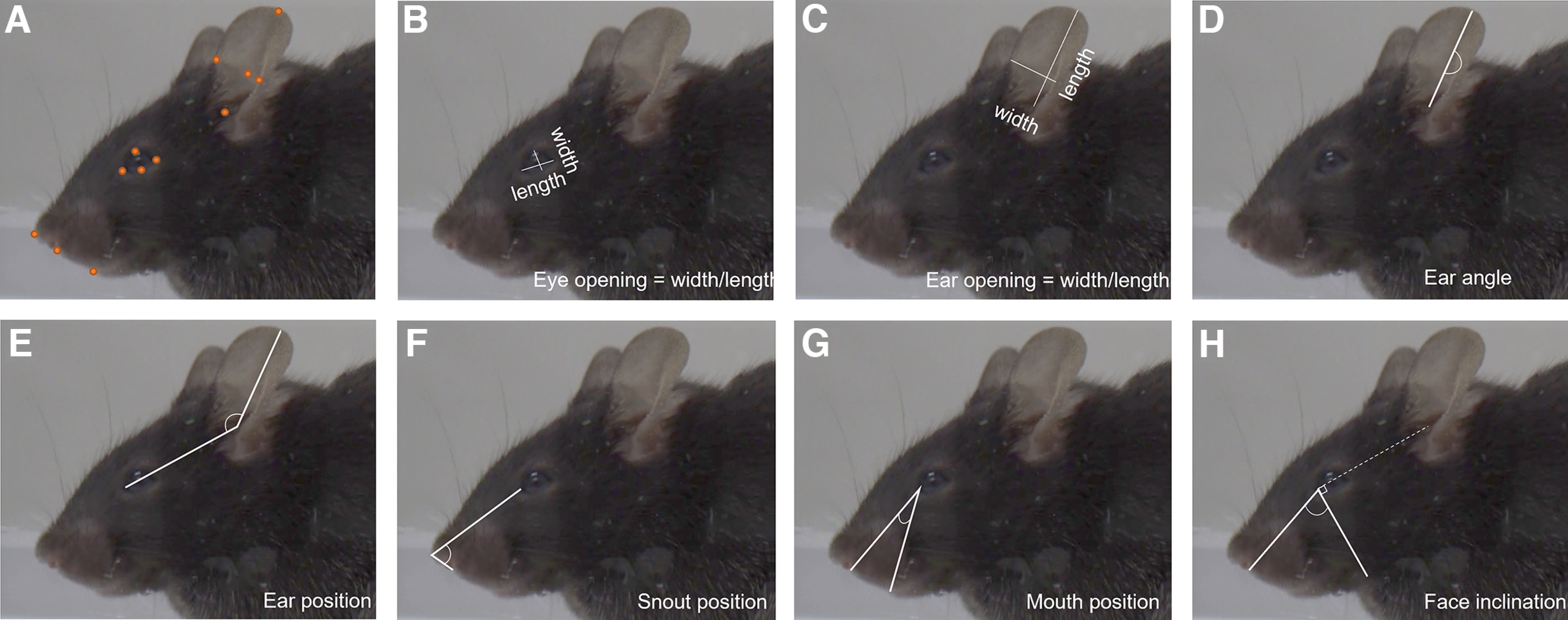
Parameters retained for facial expression analysis. ***A***, Easily visualizable facial points on mouse profile. ***B***, Eye opening. Larger values correspond to a rounder, more open eye, a ratio of 1 corresponded to a perfectly round eye. ***C***, Ear opening. Larger values correspond to the ear being placed side way, a ratio approaching 1 meaning a largely deployed and clearly visible ear pavilion. Smaller values indicate the ear being positioned higher up the head and thus a less visible ear pavilion. ***D***, Ear angle. Decreased values refer to the ear being tilted instead of straight. ***E***, Ear position. Larger values indicate the ear positioned backward. ***F***, Snout position. Smaller values indicate a pointier snout and larger values a rounder one. ***G***, Mouth position. Smaller values correspond to the bottom lip being brought forward. ***H***, Face inclination. This parameter is measured as the angle between lower snout, corner of the eye and ear orifice, to which 90° are subtracted. Smaller values indicate a more convex profile.

The review of rodent facial expression studies in the literature yielded to the identification of facial regions consistently altered by experimental procedures, including the ears, eyes and muzzle (Langford et al., 2010; Finlayson et al., 2016; Andresen et al., 2020; Dolensek et al., 2020). In turn, we identified several easily visualizable points on mouse lateral views, which allow the precise quantification of changes in these areas. Several points of reference were tested to measure mouth-, snout-related and ear-related changes, including a measure of the visible area of the inner ear. Eventually, the reference points used for measuring each facial parameter were selected to maximize the capture of facial changes and limit parameter redundance. Therefore, following extensive measurements, we retained seven facial parameters that capture quantitative changes associated with facial displays: eye opening, ear opening, ear angle, ear position, mouth position, snout position and face inclination (Fig. 1). Retained parameters included only ratios and angles, to prevent confounds such as camera zoom or individual size.

### Data collection

#### Frame extraction

Screenshots were manually extracted from video clips using VLC media player (https://www.videolan.org/). As many clear profile frames as possible were captured in each video. With regards to the tastants (water, sucrose, mentholatum, quinine), screenshots were captured exclusively when the mice had tasted the solutions, and in the 5 s following it, to avoid for taste fading. Pictures in which the mice were displaying a behavior such as licking the pipet or self-scratching were excluded, in order not to confound the facial changes elicited by the stimuli with voluntary mechanical movements. For the formalin test, a screenshot was captured every minute along the 30-min baseline and the 30-min experimental phase, resulting in 60 frames per individual.

#### Facial parameter measurements and interobserver reliability

All selected frames were imported into IC Measure (https://www.theimagingsource.com/), and the defined facial parameters were manually measured on each frame and reported into a master sheet. Measurements were made by five independent observers. All seven facial parameters retained were measured by an experienced observer on an initial selection of 144 frames corresponding to five individuals going through the SMP experiment. Two naive observers then measured the same facial parameters, resulting in good to excellent interobserver reliability assessed by intraclass correlation coefficient (ICC), ranging from 0.801 for ear angle to 0.907 for ear position. Only snout position showed moderate reliability with a coefficient of 0.655. In all other experiments, the observers were blind to the stimulation during measurements.

### Data preparation

The measures extracted from all frames related to one individual at baseline or during stimulations were averaged, to obtain a single value per stimulus, per individual. Consequently, an individual data point was based on an average of 4.96 measurements. In addition, for each stimulus for each individual, the proportional change from baseline was calculated with the formula: (value at stimulus – value at baseline)/value at baseline. Animals that belonged to different experiments but had been subjected to the same stimulation were grouped together when assessing the effect of that stimulation. Stimulations were then divided between gustatory tastants, and cutaneous stimuli: poking, petting, brushing. The formalin test and the optogenetic test were analyzed separately.

In the formalin test, response profiles are based on the entire 30 min of baseline or saline injection, as preliminary data inspection showed no changes in facial parameters over time in these cases. To the contrary, formalin is well known to elicit nocifensive responses in a two-phase pattern (Dubuisson and Dennis, 1977). Thus, the first 5 min following injection were used to constitute the early phase of formalin effect, and the 10-min interval between 20 and 30 min following injection was used for the late phase.

For the temporal analysis of facial expressions during the formalin test, the data from the 30-min baseline were averaged into a single reference point. The 30-min phase following injections was separated into six 5-min intervals. Thus, the data from the 5 min corresponding to each interval were averaged to obtain a single value per interval per individual.

### Statistical analysis

#### Control for age, sex, weight, and shaving

First, we explored the link between mouse weight and age on facial parameters at all baseline values using Pearson’s correlations. Then, we tested the effect of weight and age on the proportional change from baseline independently for each given stimulus (water, sucrose, quinine, mentholatum, poking, petting, brushing, the first 5 min following formalin injection, 30 min following saline injection, and optogenetic stimulation of Het mice) with Pearson’s correlations.

We controlled for sex difference at baseline values, and again on the proportional change from baseline for each given stimulus with *t* tests. This effect was not investigated during optogenetic stimulation because of the unbalanced sex ratio (2 F vs 7 M).

Finally, we controlled for the effect of shaving patterns on facial parameter values in response to petting during in the SMP experiment, using one-way ANOVAs. The SMP experiment was the only one in which most animals presented a shaving pattern and received a cutaneous stimulus. Therefore, it was irrelevant to control for shaving effect on other experiments.

#### Changes in facial parameters in response to gustatory and somatosensory stimuli

We proceeded to analyze the responses to drinkable stimuli (water, sucrose, quinine, mentholatum) through one-way ANOVAs followed by Tukey’s *post hoc* tests. We did not account for subject repetition as not all animals were given the same stimuli.

Responses to cutaneous stimuli (poking, petting, brushing) and the effect of the habituation protocol on these responses were analyzed with two-way ANOVAs using “habituation” and “stimulus” as factors. Tukey’s multiple comparison test was used for *post hoc* analysis.

The effect of optogenetic stimulation on facial parameters was analyzed through two-way ANOVAs with “stimulus” and “genotype” as factors, with repeated measures on the “stimulus” factor, followed by the Tukey’s *post hoc* test in case of main effect of the stimulus. In case of significant interaction, the Šidák’s multiple comparison test was used.

The temporal changes in facial parameters after saline and formalin injections were assessed using one-way ANOVAs with repeated measures, followed by Dunnett’s *post hoc* test with baseline as control.

In order also to account for individual repetition across stimuli, we plotted response profiles to each stimulus using the proportional change in each facial parameter compared with its baseline value. Difference from baseline was analyzed by one-sample *t* tests, where *p*-values were adjusted with the Bonferroni correction for the number of comparisons done in the experiment (see figure legends).

Finally, we compiled a master file based on all individual values from frame averages, with respect to each stimulus (*n* = 405 units: baseline = 114; sucrose = 61, mentholatum = 35; water = 18; quinine = 17; poking = 18; petting = 63; brushing = 40; saline = 10; formalin: early phase = 10, late phase = 10; optogenetic = 9). We used principal component analysis (PCA) to assess the use of dimensions reduction and visualize the relative contribution of each facial parameters to the variance of the dataset. We determined with Horn’s parallel analysis the number of components with eigenvalues superior to 1 after adjustment by comparison with a random dataset generated using Monte-Carlo simulation. Then, we visualized stimulus characterization based on facial expression using the uniform manifold approximation and projection (UMAP) algorithm to compute two-dimensional embeddings for each individual mouse average. We used default hyperparameters for our projection, calculating the Euclidean distance between each pair of individual averages, considering a minimum distance of 0.1 between points, and 15 neighbors.

Data analysis was conducted with GraphPad Prism 9 and R 4.1.3, using the irr, ggplot2, tidyverse, PerformanceAnalytics, FactoMineR, factoextra, paran, umap, and scales packages.

### Data availability

Data are available on reasonable request.

## Results

### Age, weight, sex and shaving pattern

First, we tested the influence of weight on absolute values at baseline for each facial parameter, all experiments pooled. We found a moderate correlation between weight and ear position, with heavier mice having ears more backward (Pearson *t*_(119)_ = 2.24, *p *=* *0.027, ρ = 0.20). Weight also negatively correlated with snout position (Pearson *t*_(119)_ = −3.20, *p *=* *0.002, ρ = −0.28), the snout being pointier in heavier animals. No other correlations between weight and baseline values were significant (all *p*s* *>* *0.073). We explored correlations between weight and proportional change from baseline for each given stimulus and found a single correlation between mouth position and weight in Het mice during optogenetic stimulation (Pearson *t*_(7)_ = −2.70, *p *=* *0.031, ρ = −0.71). No other correlation was significant (all *p*s* *>* *0.059).

Then, we tested whether mouse age at the time of the experiment affected absolute values at baseline. Conversely to weight, mouse age was positively correlated with snout position (Pearson *t*_(119)_ = 3.32, *p *=* *0.001, ρ = 0.29). Age also mildly correlated eye opening at baseline (Pearson *t*_(119)_ = −2.50, *p *=* *0.014, ρ = −0.22). We found a significant correlation between age and the proportional change from baseline in ear opening during poking (Pearson *t*_(16)_ = 3.48, *p *=* *0.003, ρ = 0.66) and in eye opening during petting (Pearson *t*_(61)_ = 2.30, *p *=* *0.025, ρ = 0.28). Other correlations were not significant (all *p*s* *>* *0.078).

Lastly, we controlled for the effect of sex on baseline values and found no significance (all *p*s* *>* *0.124). When looking at proportional changes from baseline for each stimulus, we found a single sex effect showing larger decrease in ear angle in males than in females during poking (*t*_(2.19)_ = 13.88, *p *=* *0.046). Other stimuli did not show a sex effect (all *p*s* *>* *0.066).

Weight, age and sex showed inconsistent effects that did not appear meaningful for the present study. Therefore, these factors were not accounted for further in the analyses. Finally, we controlled for the effect of shaving. Shaved patterns did not influence absolute facial parameters values in response to petting during the SMP experiment (all *p*s* *>* *0.135).

### Water and a bitter tastant elicit opposite changes in facial parameters

#### Tastants comparison

**Figure 2.**
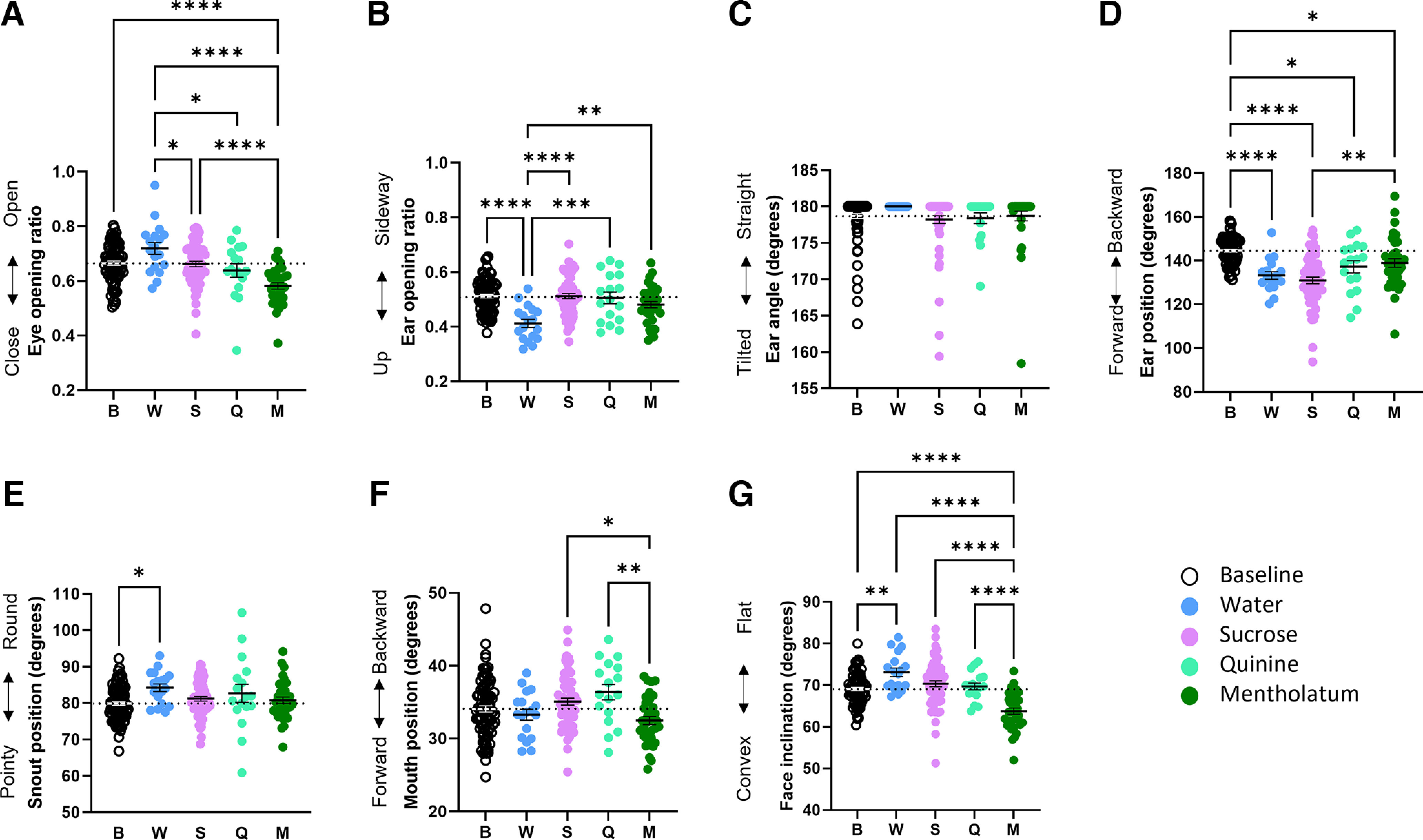
Effect of tastants on facial parameters. ***A***, Eye opening ratio. ***B***, Ear opening ratio. ***C***, Ear angle. ***D***, Ear position. ***E***, Snout position. ***F***, Mouth position. ***G***, Face inclination. Data are individual data points. One-way ANOVA followed by Tukey’s HSD. *p < 0.05, **p < 0.01, ***p < 0.001, ****p < 0.0001. Baseline, *n* = 85; water: *n* = 18; sucrose: *n* = 61; quinine: *n* = 17; mentholatum: *n* = 35. A dash line indicates the baseline value.

The only facial parameter that was not affected by the tastants was ear angle (*F*_(4,211)_ = 0.99, *p *=* *0.412). This means that the ear remained consistently straight during exposure to gustatory stimuli. We found a significant effect of the stimuli on eye opening (*F*_(4,211)_ = 11.88, *p *<* *0.001), ear opening (*F*_(4,211)_ = 9.47, *p *<* *0.001), ear position (*F*_(4,211)_ = 19.87, *p *<* *0.001), snout position (*F*_(4,211)_ = 2.95, *p *=* *0.021), mouth position (*F*_(4,211)_ = 4.39, *p *=* *0.002), and face inclination (*F*_(4,211)_ = 17.45, *p *<* *0.001). Tasting familiar water and bitter mentholatum solution elicited the strongest and most opposite facial changes (Fig. 2).

Drinking mentholatum decreased eye opening compared with baseline (*p *<* *0.001), as well as water (*p *<* *0.001) and sucrose (*p *<* *0.001). Drinking water did not modify eye opening in comparison to baseline (*p *=* *0.054), but increased it compared with sucrose (*p *=* *0.045) and quinine (*p *=* *0.016; Fig. 2*A*). Ear opening was only modified by water drinking, during which it was reduced compared with baseline and all other stimuli (all *p*s* *<* *0.005), corresponding to the ear being placed up on the head (Fig. 2*B*). Ear position was reduced by all stimuli compared with baseline (all *p*s* *<* *0.030), which corresponds to the ear pointing forward. In addition, sucrose brought the ear further forward than mentholatum did (*p *=* *0.001; Fig. 2*D*). Only water showed an effect on snout position, producing a rounder snout compared with baseline (*p *=* *0.019; Fig. 2*E*). No stimulus caused a mouth position different from baseline (all *p*s* *>* *0.149). However, sucrose and quinine increased mouth position values, positioning the mouth backward, compared with mentholatum (*p *=* *0.011 and *p *=* *0.005, respectively; Fig. 2*F*). Finally, drinking water increased face inclination values compared with baseline (*p *=* *0.005) producing a flatter face profile, whereas mentholatum decreased them, producing a more convex face, compared with baseline and all other stimuli (all *p*s* *<* *0.001; Fig. 2*G*). We found no other effects of the tastants on facial parameters (all *p*s* *>* *0.054).

#### Response profiles to tastants

**Figure 3.**
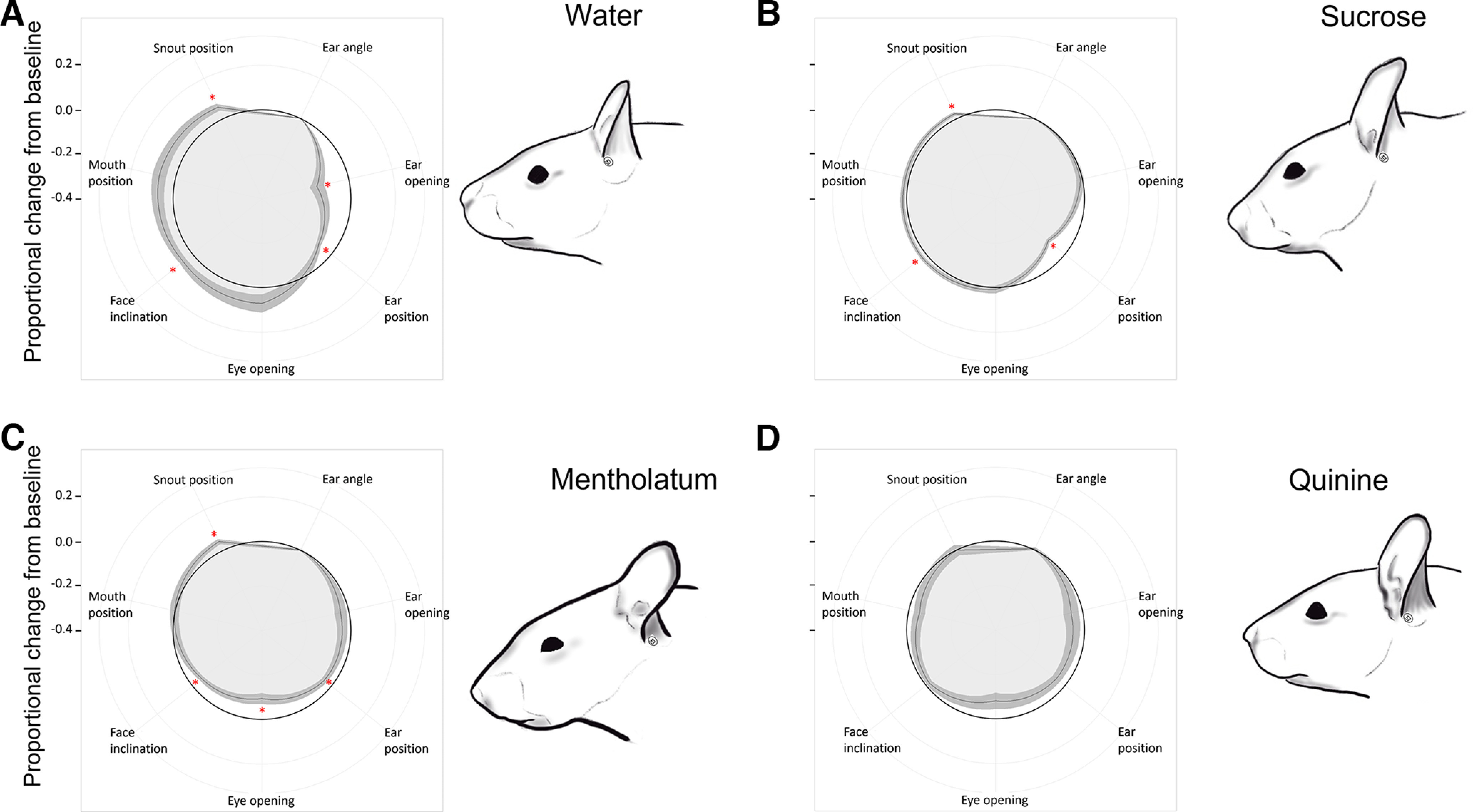
Response profiles to tastants and visual representation of the typical facial expression associated with each. ***A***, Response profile to water; *p <* *0.05, reported values are adjusted with the Bonferroni correction for three comparisons. ***B***, Response profile to sucrose; *p <* *0.05, reported values are adjusted with the Bonferroni correction for two comparisons. ***C***, Response profile to mentholatum; *p <* *0.05, reported values are adjusted with the Bonferroni correction for two comparisons. ***D***, Response profile to quinine; *p <* *0.05, reported values are adjusted with the Bonferroni correction for two comparisons. Data are mean ± SEM. One-sample *t* tests. Water: *n* = 18; sucrose: *n* = 61; quinine: *n* = 17, mentholatum: *n* = 35. Drawings are based on a quantitatively representative picture among those used for measurements. Drawings by OLM.

Responses profiles are summarized in Figure 3. Drinking water proportionally flattened the face (*t*_(17)_ = 3.18, *p *=* *0.016) and rounded the snout compared with baseline (*t*_(17)_ = 3.34, *p *=* *0.012), while it positioned the ear up and pointing forward (*t*_(17)_ = 4.59, *p *=* *0.001 and *t*_(17)_ = 6.13, *p *<* *0.001, respectively; Fig. 3*A*). Similarly, but to a lesser extent, sucrose proportionally flattened the face (*t*_(60)_ = 2.54, *p *=* *0.027) and rounded the snout (*t*_(60)_ = 2.76, *p *=* *0.015), together with bringing the ear forward (*t*_(60)_ = 9.72, *p* < 0.001; Fig. 3*B*). The mentholatum solution proportionally made the face more convex (*t*_(34)_ = 4.68, *p *<* *0.001), brought the ear forward (*t*_(34)_ = 2.96, *p *=* *0.011), closed the eye (*t*_(34)_ = 3.88, *p *=* *0.001), and rounded the snout (*t*_(34)_ = 3.48, *p *=* *0.003; Fig. 3*C*). Quinine, which at the concentration used was deemed by the experimenter to be less bitter than the mentholatum solution, showed no significant proportional change from baseline but induced similar tendencies as mentholatum in eye opening (*t*_(17)_ = 2.22, *p *=* *0.082) and ear position (*t*_(17)_ = 2.39, *p *=* *0.059; Fig. 3*D*). Other proportional changes in facial parameters did not significantly differ from baseline (all *p*s* *>* *0.136).

### Painful and innocuous somatosensory stimuli differentially affect facial parameters

#### Response profiles to mechanical somatosensory stimuli

**Figure 4.**
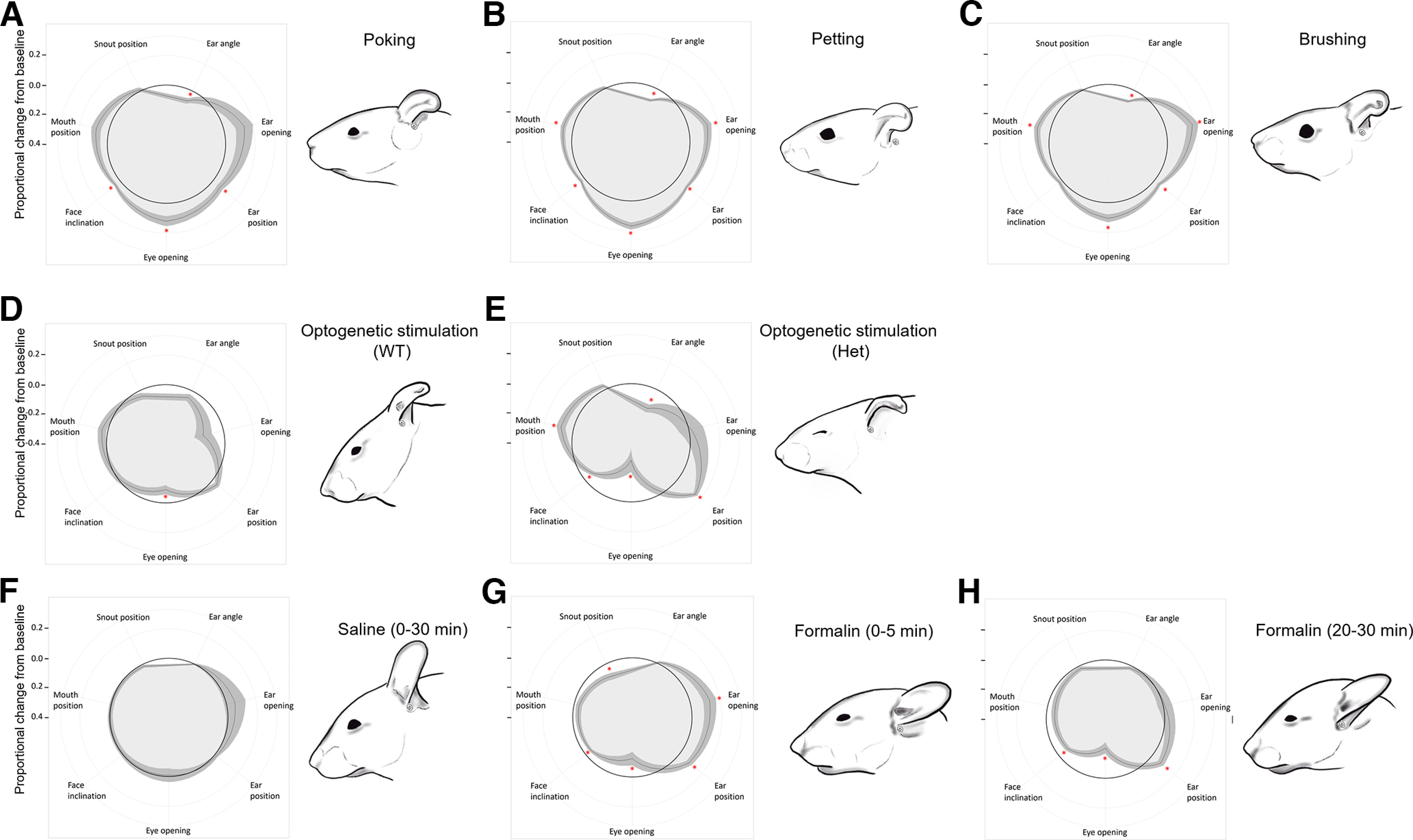
Response profiles to somatosensory stimuli and visual representation of the typical facial expression associated with each. ***A***, Poking; *p <* *0.05, reported values are adjusted with the Bonferroni correction for three comparisons. ***B***, Petting; *p <* *0.05, reported values are adjusted with the Bonferroni correction for three comparisons. ***C***, Brushing; *p <* *0.05, reported values are adjusted with the Bonferroni correction for three comparisons. ***D***, Optogenetic stimulation of the neck in WT mice; *p <* *0.05. ***E***, Optogenetic stimulation of the neck in Het mice; *p <* *0.05. ***F***, Saline injection into the hind paw (30-min average); *p <* *0.05. ***G***, Early phase of formalin injection into the hind paw (0–5 min postinjection); *p <* *0.05. ***H***, Late phase of formalin injection into the hind paw (20–30 min postinjection); *p <* *0.05. Data are mean ± SEM. One-sample *t* tests. Poking: *n* = 18; petting: *n* = 63; brushing: *n* = 40; optogenetic WT: *n* = 7; optogenetic Het: *n* = 9; saline: *n* = 10; formalin early and late phases: *n* = 10. Drawings are based on a quantitatively representative picture among those used for measurements. Drawings by OLM.

Response profiles are based on pooled data of naive and touch-habituated mice. Poking the mice on the neck produced a tilted ear compared with baseline (*t*_(17)_ = 3.66, *p *=* *0.006), associated with an ear placed backwards (*t*_(17)_ = 2.79, *p *=* *0.038), a larger eye opening (*t*_(17)_ = 3.50, *p *=* *0.008) and a flattened face (*t*_(17)_ = 3.79, *p *=* *0.003; Fig. 4*A*). Petting and brushing the mice in the same area produced a similar response profile as poking, tilting the ear (*t*_(62)_ = 8.60, *p *<* *0.001 and *t*_(39)_ = 7.89, *p *<* *0.001, respectively), placing it backwards (*t*_(62)_ = 8.57, *p *<* *0.001 and *t*_(39)_ = 3.50, *p *=* *0.004, respectively) and increasing eye opening (*t*_(62)_ = 6.86, *p *<* *0.001 and *t*_(39)_ = 3.94, *p *=* *0.001, respectively). The face was also flattened in the case of petting (*t*_(62)_ = 2.66, *p *=* *0.030) but not of brushing (*t*_(39)_ = 1.58, *p *=* *0.364). Instead, both petting and brushing resulted in the ear being placed on the side of the head (all *p*s* *<* *0.001), a pattern not significant when poking the mice (*t*_(17)_ = 2.33, *p *=* *0.097), together with the mouth being positioned backward too (all *p*s* *<* *0.002), which was also nonsignificant during poking (*t*_(17)_ = 2.53, *p *=* *0.065; Fig. 4*B*,*C*). Cutaneous stimuli did not produce other significant proportional changes from baseline in facial parameters (all *p*s* *>* *0.364).

Optogenetic stimulation of the nape of the neck in WT mice only significantly reduced eye opening (*t*_(6)_ = 2.99, *p *=* *0.024; Fig. 4*D*). All other parameters were unaffected (all *p*s* *>* *0.059). To the contrary, in Het mice, optogenetic stimulation reduced eye opening (*t*_(8)_ = 3.59, *p *=* *0.007), tilted the ear (*t*_(8)_ = 4.82, *p *=* *0.001), brought the ear and the mouth backward (ear position: *t*_(8)_ = 9.13, *p *<* *0.001; mouth position: *t*_(8)_ = 2.55, *p *=* *0.034), and made the face more convex (*t*_(8)_ = 4.00, *p *=* *0.004). Ear opening and snout position were unaffected (all *p*s* *>* *0.123; Fig. 4*E*).

Mice injected with saline in the hind paw showed no proportional facial change from baseline (all *p*s* *>* *0.217; Fig. 4*F*). During the first 5 min following formalin injection into the hind paw, we observed a smaller eye opening (*t*_(9)_ = 2.77, *p *=* *0.022), the ear being placed sideway and backwards (ear opening: *t*_(9)_ = 2.94, *p *=* *0.016; ear position: *t*_(9)_ = 3.93, *p *=* *0.004), a pointier snout (*t*_(9)_ = 3.94, *p *=* *0.003) and a more convex face (*t*_(9)_ = 4.39, *p *=* *0.002; Fig. 4*G*). During the late phase of the formalin test, three of the five changes observed during the first phase were conserved: the eye was more closed (*t*_(9)_ = 5.38, *p *<* *0.001), the ear positioned backward (*t*_(9)_ = 3.21, *p *=* *0.011) and the face more convex (*t*_(9)_ = 4.68, *p *=* *0.001; Fig. 4*H*).

#### Habituation to light touch only modestly modulates facial responses

There was a main effect of cutaneous stimuli on all facial parameters measured but snout position: eye opening (*F*_(3,198)_ = 19.69, *p *<* *0.001), ear opening (*F*_(3,198)_ = 9.30, *p *<* *0.001), ear angle (*F*_(3,198)_ = 33.74, *p *<* *0.001), ear position (*F*_(3,198)_ = 24.16, *p *<* *0.001), snout position (*F*_(3,198)_ = 2.53, *p *=* *0.059), mouth position (*F*_(3,198)_ = 8.40, *p *<* *0.001), and face inclination (*F*_(3,198)_ = 5.82, *p *=* *0.001). We found no interaction between habituation and stimuli for any of the parameters (all *p*s* *>* *0.077).

**Figure 5.**
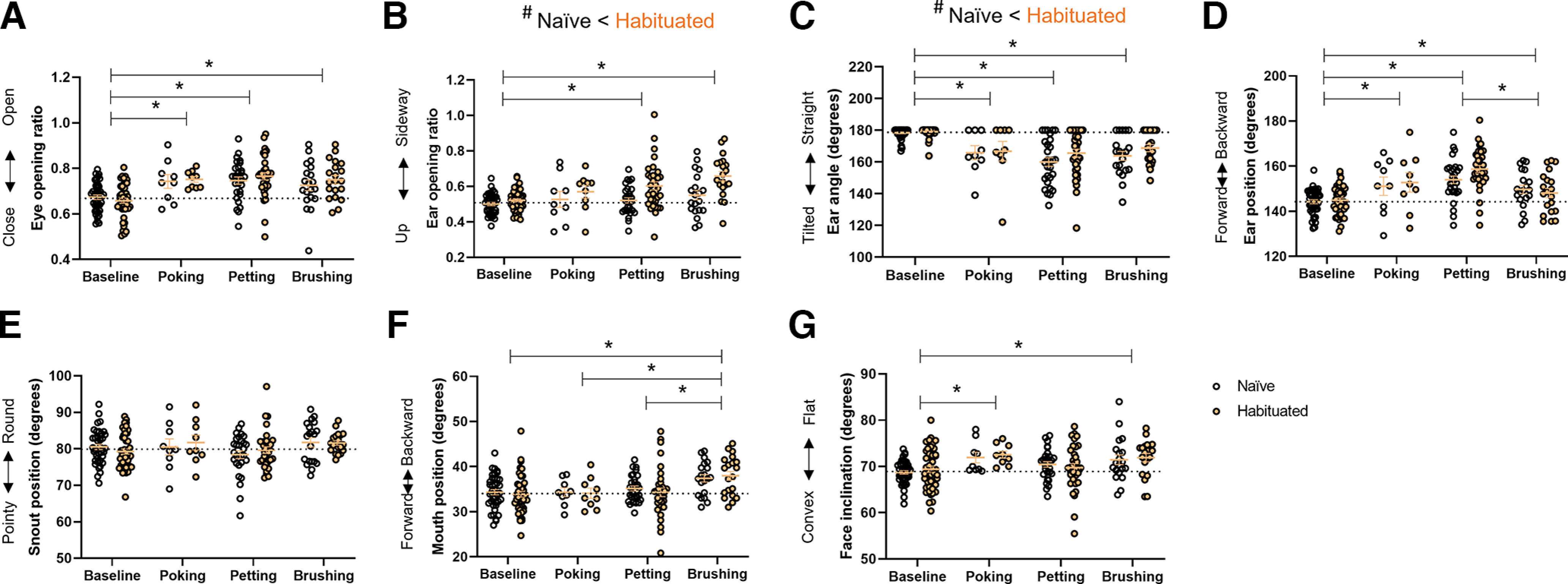
Effect of habituation on cutaneous stimuli. ***A***, Eye opening ratio. ***B***, Ear opening ratio. ***C***, Ear angle. ***D***, Ear position. ***E***, Snout position. ***F***, Mouth position. ***G***, Face inclination. Data are individual data points. A dash line indicates the average baseline value. Two-way ANOVA. Stimulus effect: *p <* *0.05. Main habituation effect: #*p *<* *0.05. Baseline: *n* = 40 naive + 45 habituated; poking: *n* = 9 naive + 9 habituated; petting: *n* = 29 naive + 34 habituated; brushing: *n* = 20 naive + 20 habituated.

Poking, petting, and brushing the mice all increased eye opening compared with baseline (all *p*s* *<* *0.001; Fig. 5*A*). The ear was placed sideway during petting (*p* = 0.010) and brushing (*p *<* *0.001), but not poking (*p *=* *0.59; Fig. 5*B*). All cutaneous stimuli tilted the ear compared with baseline (all *p*s* *<* *0.001; Fig. 5*C*). The same pattern was observed on ear position, with all cutaneous stimuli moving the ear backwards (all *p*s* *<* *0.012; Fig. 5*D*). Petting the mice elicited the most backward ear position, significantly more than during brushing (*p *=* *0.001; Fig. 5*D*). Brushing moved the mouth backward compared with baseline (*p *<* *0.001) and to other cutaneous stimuli (poking, *p *=* *0.009; petting, *p *=* *0.001; Fig. 5*F*). Finally, poking and brushing the mice, but not petting them (*p* =0.400), flattened the face compared with baseline (poking-baseline, *p *=* *0.012; brushing-baseline, *p *=* *0.004; Fig. 5*G*).

We found a moderate effect of the habituation protocol. Habituation increased the pattern of ear opening caused by cutaneous stimuli (*F*_(1,198)_ = 15.98, *p *<* *0.001; Fig. 5*B*). In parallel, habituation attenuated the ear tilt caused by poking, petting, and brushing (*F*_(1,198)_ = 6.10, *p *=* *0.014; Fig. 5*C*).

### Facial expressions convey pain-associated affects in real time

#### Optogenetic stimulation of CGRP-lineage sensory fibers

**Figure 6.**
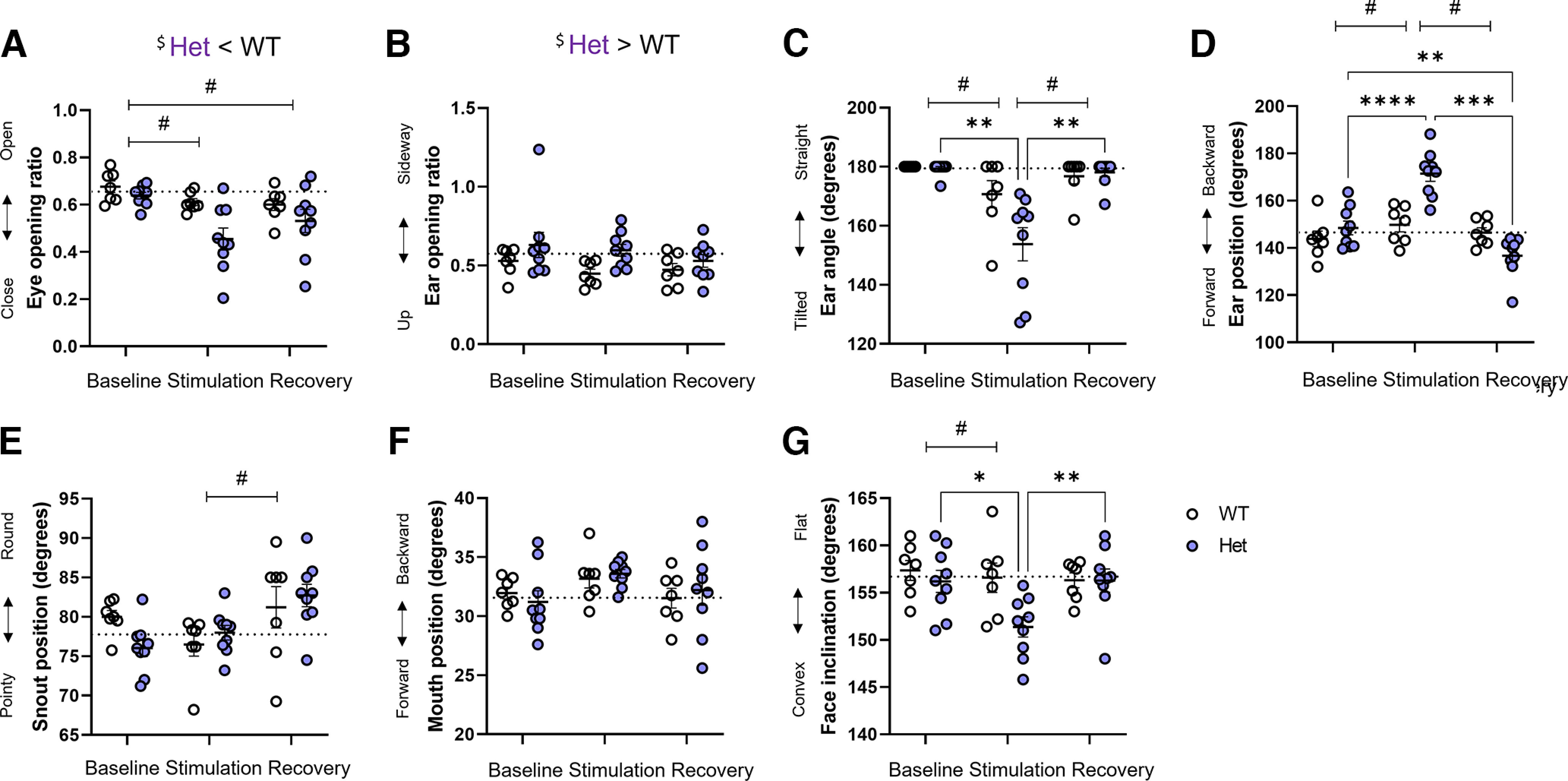
Effect of optogenetic stimulation of the nape of the neck on CGRP;LSL-ReaChR mice. ***A***, Eye opening ratio. ***B***, Ear opening ratio. ***C***, Ear angle. ***D***, Ear position. ***E***, Snout position. ***F***, Mouth position. ***G***, Face inclination. Data are individual data points. A dash line indicates the average baseline value. Repeated measures two-way ANOVAs. Interaction between stimulus and genotype: *p <* *0.05, **p <* *0.01, ***p <* *0.001, ****p <* *0.0001. Main stimulus effect: #*p *<* *0.05. Main genotype effect: $*p *<* *0.05. WT: *n* = 7; Het: *n* = 9.

We found an interaction between genotype and optogenetic stimulation on ear angle (*F*_(2,28)_ = 5.91. *p *=* *0.007), ear position (*F*_(2,28)_ = 23.10, *p *<* *0.001), and face inclination parameters (*F*_(2,28)_ = 4.78, *p *=* *0.016). Het, but not WT mice, had a tilted ear during stimulation compared with baseline and recovery periods (Het: stimulation-baseline: *p *=-0.004; stimulation-recovery: *p *=* *0.005; WT: all *p*s* *>* *0.167; Fig. 6*C*). Similarly, only Het mice showed a backward ear position during optogenetic stimulation compared with both baseline (*p *<* *0.001) and recovery period (*p *<* *0.001). Furthermore, Het mice showed a more forward ear position during recovery than at baseline (*p *=* *0.005; Fig. 6*D*). Finally, Het mice had a more convex face profile during optogenetic stimulation compared with baseline (*p *=* *0.012) and recovery (*p *=* *0.010; Fig. 6*G*).

These interactions were accompanied by a main effect of the test period on eye opening (*F*_(1.70,23.83)_ = 9.11, *p *=* *0.002), ear angle (*F*_(1.26,17.66)_ = 21.26, *p *<* *0.001), ear position (*F*_(1.38,19.27)_ = 36.68, *p *<* *0.001), snout position (*F*_(1.66,23.24)_ = 7.43, *p *=* *0.005), and face inclination (*F*_(1.84,25.74)_ = 5.74, *p *=* *0.010). Both Het and WT mice had reduced eye opening during optogenetic stimulation and recovery compared with baseline (stimulation-baseline: *p *=* *0.003; recovery-baseline: *p *=* *0.037; Fig. 6*A*). Both had the ear more tilted during stimulation than during baseline (*p *=* *0.001) and recovery (*p *=* *0.002; Fig. 6*C*), and the ear more backward during stimulation than during baseline (*p *<* *0.001) and recovery (*p *=* *0.002; Fig. 6*D*). The snout appeared pointier during stimulation than recovery (*p *=* *0.004; Fig. 6*E*), and the face more convex during stimulation than at baseline (*p *=* *0.041) for both Het and WT mice (Fig. 6*G*).

We found two simple effects of mouse genotype on facial parameters, showing slightly smaller eye opening in Het than WT (*F*_(1,14)_ = 6.25, *p *=* *0.025) and ears placed more sideway in Het than WT mice (*F*_(1,14)_ = 4.82, *p *=* *0.046; Fig. 6*A*,*B*). We found no other effect of genotype nor of optogenetic test, and no further interaction between these factors (all *p*s* *>* *0.074).

#### Formalin test

**Figure 7.**
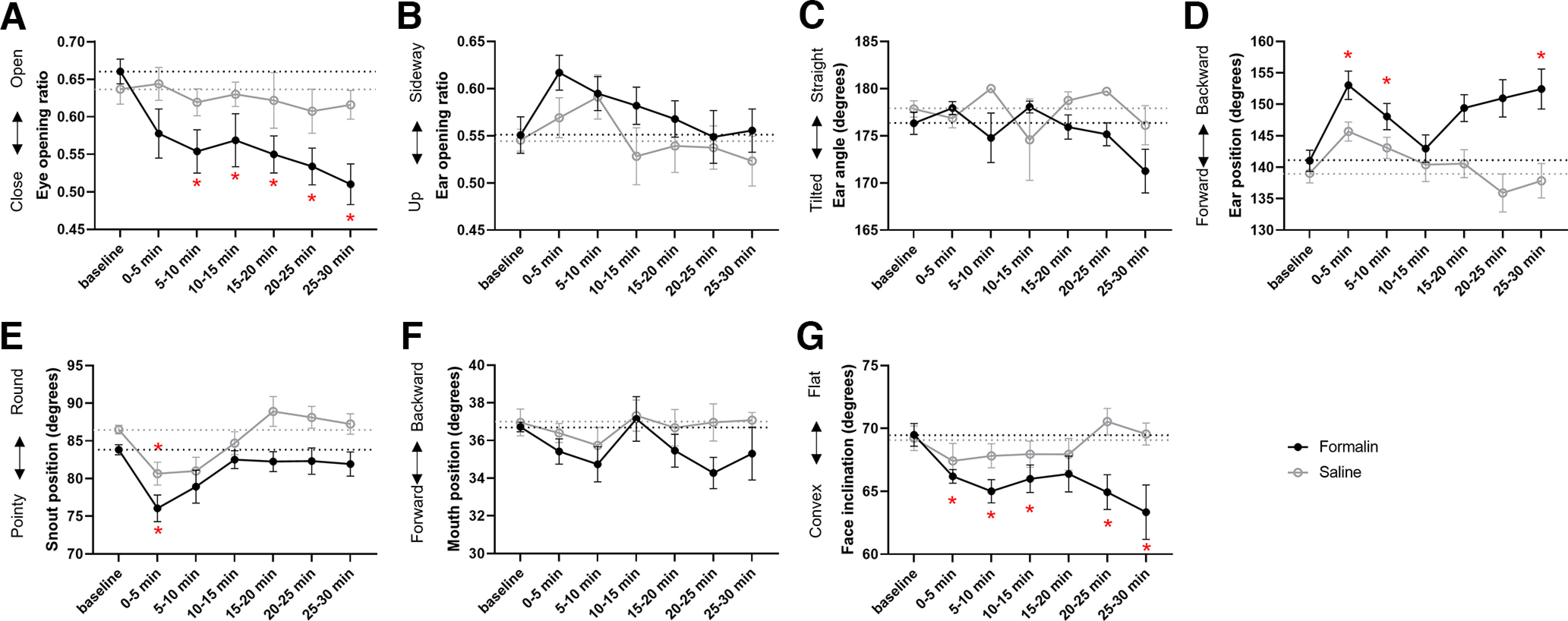
Real-time effect of saline or formalin injections into the hind paw on facial parameters. ***A***, Eye opening ratio. ***B***, Ear opening ratio. ***C***, Ear angle. ***D***, Ear position. ***E***, Snout position. ***F***, Mouth position. ***G***, Face inclination. Data are mean ± SEM; *p <* *0.05, difference from the corresponding baseline. Saline: *n* = 10; formalin: *n* = 10.

First, we assessed the temporal dynamics of facial changes across a 30 min period following saline injection into the hind paw compared with a preinjection baseline period. We found very limited effects of saline. There was a main effect of the injection on ear position (*F*_(4.05,36.49)_ = 3.25, *p *=* *0.022), but *post hoc* tests did not reach significance (all *p*s* *>* *0.072; Fig. 7*D*). There was also a main effect of saline injection on snout position (*F*_(3.11,27.95)_ = 4.93, *p *=* *0.007), with *post hoc* tests revealing that the snout was pointier during the first 5 min following injection than at baseline (*p *=* *0.043; Fig. 7*E*). We found no other effect of saline on facial parameters (all *p*s* *>* *0.206).

Second, we conducted the same analysis after formalin injection into the hind paw. There was no effect of formalin injection on ear opening, ear angle nor mouth position (all *p*s* *>* *0.081; Fig. 7*B*,*C*,*F*). However, the formalin assay modified eye opening over time (*F*_(2.6.6,23.90)_ = 4.24, *p *=* *0.018). We found a steady reduction in eye opening following formalin injection compared with baseline, from 5 min postinjection to the end of the test (all *p*s* *<* *0.030; Fig. 7*A*). Formalin injection temporally affected ear position (*F*_(3.57,32.11)_ = 5.24, *p *=* *0.003). The ear moved backward during the first 10 min following injection (all *p*s* *<* *0.047), and again between 25 and 30 min following injection (*p *=* *0.044; Fig. 7*D*). Similarly to saline, formalin modified snout position (*F*_(3.17,28.54)_ = 3.19, *p *=* *0.036). The snout was pointier during the first 5 min following formalin injection compared with baseline (*p *=* *0.014; Fig. 7*E*). Formalin injection strongly modified face inclination (*F*_(3.75,33.79)_ = 4.18, *p *=* *0.009). The face was consistently more convex during the first 15 min following formalin injection (all *p*s* *<* *0.028), and again between 20 and 30 min following it (all *p*s* *<* *0.017; Fig. 7*G*).

### Facial landscape of emotional stimuli

**Figure 8.**
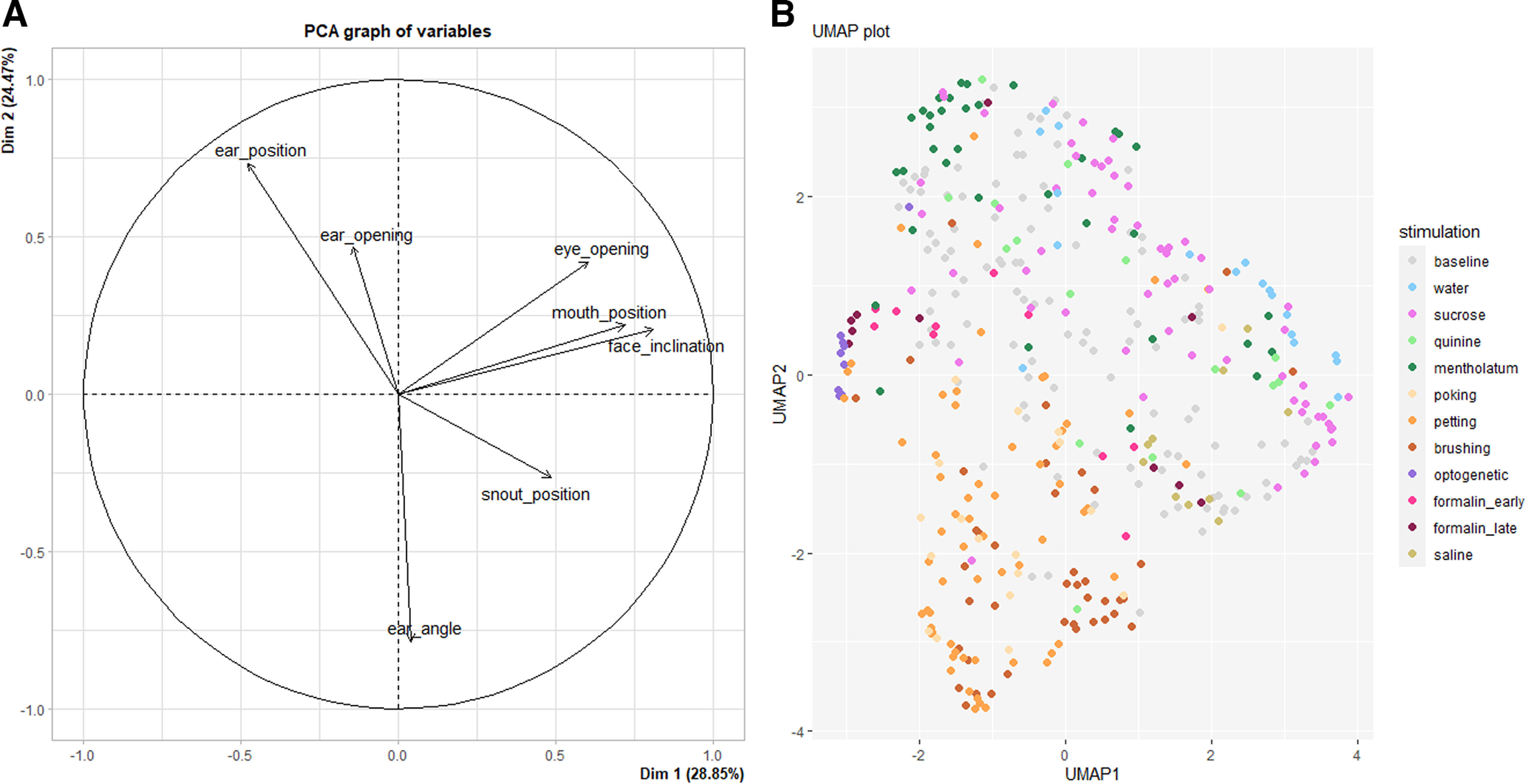
***A***, Principal component analysis of mouse facial parameters across 11 stimuli (counting early and late formalin phases separately) and their respective baselines. The first component (Dim. 1) is interpreted as Eye and Muzzle movement, and the second (Dim. 2) as ear-related movements. ***B***, Visualization with UMAP of individual average values in response to 10 stimuli and at baseline. *n* = 405 units. Metrics = Euclidean, Neighbors = 15. Minimum distance = 0.1.

Facial parameters were reduced by PCA producing a total of seven dimensions. Following Horn’s parallel analysis, two dimensions had adjusted eigenvalues >1, cumulatively explaining 53.32% of the variance. The first dimension, to which contributed mostly face inclination (32.38%), mouth position (25.60%) and eye opening (17.98%), accounted for 28.85% of the variance and can be interpreted as eye and muzzle movements. The second dimension, to which contributed mostly ear position (31.41%) and ear angle (36.04%) explained 24.47% of the variance and can be interpreted as ear-related movement (Fig. 8*A*).

Since the number of dimensions produced by PCA was the same as the initial number of measured facial parameters, and since the two principal components only explained 53.32% of the variance, we used UMAP directly on absolute measured values to visualize clusters of stimuli. The projection based on 405 units made of individual averages in response to each stimulus produced a clear but partly overlapping clusterization. Labeling revealed an aggregation of units related to mechanical cutaneous stimuli (poking, petting, brushing). Optogenetic stimulation clustered in close proximity with many units related to the early and late phases of formalin action after injection. Gustatory stimuli appeared visibly separated, with a distinction between mentholatum and sucrose/water (Fig. 8*B*).

## Discussion

Animal emotions have attracted a lot of attention in the context of improving animal welfare and compensating the reductionist bias that has long prevailed in behavioral neuroscience. In this study, the easily visualizable points defined on mouse profiles allowed the fine quantification of facial changes and successfully captured different profiles in response to aversive and pleasurable gustatory and somatosensory stimuli.

### Summary of the results

Drinkable solutions, independently from their inherent valence, produced a common ear movement forward. This might be a confound of the drinking movement despite efforts made to reduce this effect. However, our most salient stimuli, water and mentholatum, oppositely influenced eye opening and face inclination, suggesting that they are reliable indicators of a gustatory stimulus’ valence. Quinine’s lack of significant effect might be attributed to its comparatively lower bitterness compared with mentholatum.

All effective somatosensory stimuli proportionally positioned the ear more backward than at their respective baseline. We suggest this is a protective reflex of the sensitive ears elicited by any type of somatosensory stimuli. The often observed decrease in ear angle producing an ear tilt might participate in ear protection too. Overall, light somatosensory stimuli including poking, petting, and brushing tended to increased eye opening and face inclination values, all the while painful formalin injections and optogenetic stimulation of CGRP-lineage fibers had opposite effects on these parameters. WT animals showed some degree of response during optogenetic stimulation, mostly related to eye closing, and likely constituted by general light avoidance behavior and the presence of the optic fiber above their neck. Poking the mice with a stationary stimulus elicited a very similar pattern as petting and brushing them at 5 cm/s. Brushing the mice produced the highest facial changes, in particular positioning the mouth more backward. During both petting and brushing, the ears appeared turned toward the side of the head, a pattern further increased by habituation.

Formalin injection modified facial parameters in a clear two-phase pattern, following its well-known two-phase effect on nocifensive behavior. The only common effect between saline and formalin injection were a decrease in snout position values in the first 5 min following injection, which might be a confound of isoflurane anesthesia (Miller et al., 2015; Hohlbaum et al., 2017).

### Methods validation

The new method developed here proposes to rigorously measure the ratios and angles visible on a mouse profile. This allowed some independence from observer training, as measures were objective, based on easily visualizable points on the face. Calculating proportional changes from baseline ranged from an average −36% to +22% change in facial parameters in response to the different stimuli, with the most prominent changes usually occurring in eye opening and ear position. The absence of restraint in our study prevented the confound of restraint stress which was shown to increase MGS scores (Senko et al., 2017). Multiple observers scored the present data; all data accurately showed similar patterns across stimuli and across experiments, regardless of the observer. Several sequences of stimuli were used to elicit a range of affective states, along both gustatory and somatosensory modalities. We did observe that the sensory modality stimulated influenced the pattern of facial changes, further pointing the relevance of including several modalities in a stimulation sequence to account for potential confounds.

We included seven facial parameters, which showed some degree of intercorrelation (e.g., proportional change from baseline in ear angle strongly correlated that of ear position, all experiments collapsed: Pearson *t*_(279)_ = −10.35, *p* < 0.001, ρ = −0.53, all stimuli collapsed). This is consistent with the fact that some of the angles and ratios measured could have facial points in common. Yet, the first two components produced by PCA retained only 53.32% of the data variance. Moreover, the differential changes observed in facial parameters in response to different stimuli (e.g., clear backward movement of the mouth during petting, brushing and optogenetic stimulation absent in response to gustatory stimuli) argues in favor of retaining all seven. Especially, this seems relevant if this method were to be applied to other mammal species in which parameters correlation and mobility might vary because of different facial morphology and musculature.

### Salient facial features in the mouse produce robust response profiles

Combining the seven facial parameters, we were able to determine response profiles to each stimulation. Some stimuli elicited similar response profiles, such as petting and brushing the mice at similar pressure and velocity. To the contrary, some facial parameters showed bidirectional changes in response to stimuli at the opposite ends of the pleasantness range. The quantification of facial changes and their proportional comparison to baseline values support the ability for facial expression analysis to detect positive versus negative affects, in combination with the modality stimulated.

The dataset used in this study encompassed more facial parameters than the initial MGS, allowing to precisely determine which facial components contribute to the scores attributed in the MGS (Table 1). Ears are particularly mobile in rodents as well as in other species, and thought to both serve social communication and reflect internal states such as arousal or vigilance (Lecorps and Féron, 2015), fear and aggression (Defensor et al., 2012), pain (Andresen et al., 2020), or possibly positive emotional valence (Descovich et al., 2017). Ear-related parameters were consistently affected by the different stimuli, suggesting that ears might be a critical feature in emotional communication. This is consistent with the large size of the ears relatively to the mouse face that would make them an evolutionary relevant tool for social communication in rodents. This substantially differs from human models in which ear movement is limited, although not absent, and ears themselves relatively small and often concealed under hair. Nevertheless, the parameters defined in this study are applicable to most mammal species. Thus, the conservation of facial movements across species, as well as species-specific salient features could be verified in future studies.

**Table 1:** Correspondence between facial changes identified in the original MGS (Langford et al., 2010) and the method developed in this paper

MGS items	Corresponding facial parameters	Change direction
Orbital tightening	Eye opening	Smaller eye opening
Nose bulge	Snout position Mouth position Face inclination	Pointier snout? Mouth backward More convex face
Cheek bulge	Snout position Mouth position Face inclination	Rounder snout? Mouth backward More convex face
Ear position	Ear opening Ear position Ear angle	Ear to the side Ear moved backward Tilted ear
Whisker change	Snout position Mouth position Face inclination	Rounder snout? Mouth backward More convex face

It is intuitive to think that facial changes would be more pronounced in the areas corresponding to the sensory organ detecting the stimulation. Some facial changes were likely not elicited by the subjective experience of a stimulation, but indeed simply a result of facial contact and may serve a protective function (Defensor et al., 2012). However, measures of the muzzle area were also influenced during nongustatory stimuli, and ear parameters changed although formalin was injected into the hind paw. These observations argue in favor of the changes observed being indicators of emotional valence.

### Temporal dynamics in the formalin test

Several substances can be used intradermally to induce pain. However, in freely moving mice we found that scratching the injection site might confound results as mice tend to lick their paws following scratching, thus also tasting the injected compound, which could cause oral pain in the case of capsaicin or mustard oil (allyl isothiocyanate). In addition, mustard oil caused burning-like ocular pain in the confined space of the observation cubicle (unpublished observations). In light of these considerations, we used the formalin test to induce pain intradermally. Several facial parameters changed dynamically following formalin injection, correlating with its two-phase action. Such an observation shows that facial expressions, or grimace scales, are useful tools to implement in real time as they accurately reflect instantaneous changes in internal states. It is also noteworthy that return to baseline levels was almost instantaneous after stopping painful optogenetic stimulation, further strengthening the finding that facial changes inform on spontaneous affects.

In parallel to instantaneous facial changes during the formalin assay, other parameters such as eye opening showed a stable effect across the entire postinjection period, independently from the early and late phases of formalin action. One hypothesis would be that facial expressions reflect both spontaneous responses to specific stimuli such as painful experiences, and the underlying internal states such as unfocused anxiety. Interestingly, and in accordance with previous findings using the MGS (Langford et al., 2010), the effect of formalin on eye opening was not significant during the first phase with respect to absolute values. However, the proportional decrease from baseline in eye opening was significant during both phases.

### Limitations

Several previous studies have reported facial asymmetry as an indicator of confusion, poor welfare or pain (Sefcek and King, 2007; Boissy et al., 2011; Gleerup et al., 2015). It is possible that facial asymmetry would have completed these data and further shed light on its relationship to emotional valence. Unfortunately, it was not possible to account for this variable when looking at lateral views. The present stimuli sequences aimed at inducing a range of positive and negative affects. Most importantly, we attempted to develop a new positive stimulus in the form of pleasant touch. Recently, it was shown that male mice developed conditioned place preference in response to gentle stroking of the fur (Cho et al., 2021). In the present study, some of the mice were naive to this type of stimulation and might have expressed fear of the experimenter’s fingers or of the brush. Whether we successfully induced pleasantness is unclear. We targeted the nape of the neck, which is stimulated in wild mice during social allogrooming, but experimenter observations suggest that at least some mice exhibited defensive postures. Nevertheless, in the present study, poking, petting, and brushing the mice produced similar response profiles of gradually increasing intensity. These response profiles were largely different from those elicited by optogenetic stimulation of the same zone. In order to prevent fear of the experimenter, we habituated some of the mice to cutaneous stimuli, which resulted in the accentuation of ear opening changes but in a reduction in ear angle. Finally, in our study, ear position varied similarly to the ear angle defined by Finlayson et al. (2016) during rat tickling, which was associated with vocalizations typical of hedonic situations. These elements might signal a positive affect elicited by brushing the mice.

Clustering of stimuli through facial changes was investigating using UMAP. This projection was based on a relatively small sample of 405 units, which might explain the dispersion of the results. Nevertheless, coherent unit aggregations are visible, consistent with the typical face profile elicited by the cutaneous stimuli, and the common facial changes elicited by the gustatory stimuli. Interestingly, baseline units, despite showing a high variability, were well separated from cutaneous stimuli, optogenetic stimulation and mentholatum. This further highlights the difficulty to establish indicators of positive affects as their features might not be as strongly separated from those of a neutral state. Nevertheless, based on this projection, it appears that the inclusion of new individual units from unknown situations would inform on stimulus modality and valence within that frame. In humans, subjective affect is usually rated in terms of valence and arousal. However, valence itself is ambiguous and cannot be optimally modelled by linear models, even in scale ratings (Mattek et al., 2017). We might add that, in nonverbal animal models, in which we assess purposefully triggered affects through chosen stimuli, the characteristics of the stimuli might also influence valence measurements. The method established in the present study captures detailed changes in facial expression that offers potential to be automated and generalized to multiple species and affective situations.

### Prospective machine learning

The future of neuroscience will rely on the use of machine learning. One of the pitfalls of this powerful tool, is that the opacity of this black box approach often hinders human understanding. In our study, we aimed at identifying key facial features involved in facial expression changes and validate their significance in characterizing reactions to several emotional stimuli. We found that even in mice that have a relatively less mobile facial musculature than humans, facial expressions are complex, with some parameters changing similarly in situations of opposed valence. This is not new, as the development of the facial action coding system (Ekman and Friesen, 1978) already identified action units that convey different meaning while showing the same directional change (e.g., lid tightener active in sadness and fear; Hamm et al., 2011). The meaning of a facial expression can therefore only be extracted when looking comprehensively at multiple facial parameters and their interactions, for which machine learning models are highly useful. The facial points used when measuring the hereby defined parameters are easily labelable for a pose estimation softwares like DeepLabCut or SLEAP. The use of machine learning in the future will improve the data collection process and refine emotion identification, while potentially identifying other parameters of interest in freely moving animals, based on the parameters here defined and validated

In conclusion, we report the first case of rodent facial expression analysis with a newly developed method, using several positive, non-noxious stimuli. Our new method uses easily visualizable facial points resulting in seven facial parameters, independent from camera zoom and animal size. The parameters are entirely scalable, conveying meaningful facial information of mouse subjective experience during sequences of emotion-inducing stimuli. The parameters retained are generalizable to most mammals and can be a useful tool to identify overlaps between emotions and their conservation across species. Future studies may focus on the potential for this method to be successfully translated to humans. The present study provides a transparent understanding of the facial regions responsible for the production and perception of facial expressions. These expressions have the potential to reflect spontaneous, stimulus-directed responses, together with general internal state, as well as stimulus-associated mechanical movements. As such, the sensory modality stimulated and the area of stimulation might influence the severity of facial displays.

Pleasant social touch showed potential for eliciting positive affect, and might be used in the future to mitigate pain or improve resilience from negative experience. Gentle touch delivered by an experimenter may need conditioning to be pleasant to mice, but social touch, and mechanisms such a social buffering have robustly shown rewarding value in social animals. It would be valuable in the future to automatize the capture of mouse facial expressions in a more naturalistic, socially complex model.

Even considering the increasing public concern for animal welfare, and the social imperative for personal development and happiness, positive affects are rarely addressed, and research remains biased toward the study of negative experiences (Webb et al., 2019). This bias leaves out a large panel of affects, not only offering an incomplete view of the issue of mental health, but also distorting the prism of data interpretation. We propose that including positive, ecologically relevant situations, together with improving our understanding of positive affects, will positively impact health research.

## Synthesis

Reviewing Editor: Christina Zelano, Northwestern University - Chicago Decisions are customarily a result of the Reviewing Editor and the peer reviewers coming together and discussing their recommendations until a consensus is reached. When revisions are invited, a fact-based synthesis statement explaining their decision and outlining what is needed to prepare a revision will be listed below. The following reviewer(s) agreed to reveal their identity: Sam Cooler.

Overall, reviewers agreed that this work is timely and important. However, reviewers raised concerns about the analyses included in the manuscript. Both reviewers felt that more in-depth analyses would substantially improve the proposed method. See their comments below:

Reviewer 1:

In this paper, authors present what they describe as a new approach to observing and quantifying mouse facial expressions in different contexts. Facial expressions are promising external indicators of internal states in mice which have received a considerable amount of attention over the last decade. Nonetheless, the field still lacks a generally agreed upon set of expressions and ways to quantify them, especially in freely moving mice.

Overall, this paper is timely and the experiments conducted are overall interesting. However, I believe that the facial expression analysis at the center of this work lacks in depth analysis necessary to support many of the claims made by the authors.

Major issues:

1) Attempting to quantify rodent facial expression by measuring various facial angles is not new. Previous studies (e.g. Finlayson et al., 2016, Plos One; a study not cited by the authors) have approached this question in a very similar manner and even established many, if not most, of the measurements used in this paper. I would suggest the authors carefully consider their claims of novelty and ensure they are accurately positioned in the framework of existing literature.

2) The method described in this paper would benefit from better justification. The authors suggest that the 7 measurements described in this paper were retained based on extensive measurements. Describing what were the other measurements tested and what were the thresholds for measurement retainment would provide useful information for future facial expression studies and provide support for the validity of the chosen measurements.

3) It is not very convincing that all of the 7 measurements are useful and necessary for quantifying expression. The authors state that the measurements are significantly correlated to each other. If that is the case, how many measurements are needed to actually distinguish between the stimuli presented and how many to distinguish between the underlying states? It would be interesting to see dimensionality reduction (for example using PCA) performed on the quantitative measurements. This might also help authors quantify the differences and similarities in the pattern of measurement changes across different stimuli. Currently, these comparisons are rather qualitative and in my opinion insufficient to claim that the method infers emotion states, for which much more evidence would be needed.

4) Innocuous optogenetic stimulation of WT control mice resulted in what looks like a significant change across the expression measurements. Why?

Reviewer 2:

In this work, the authors describe a new method for the evaluation of mouse facial expressions. They introduce a set of seven parameters based on the measurement of twelve points on the lateral view of a mouse, as single-frame images with manual annotation. They present the changes from the baseline of these parameters during a set of positive and negative valence gustatory and somatosensory experiments, as well as during optogenetic stimulation of somatosensory nerves. The results show some clear patterns and are presented with statistical analysis.

Overall, I find that this work presents an interesting new way to evaluate the facial expressions of mice during experiments. The experimental methods appear solid, although this is not my specific area of expertise. The amount of data appears to be enough to support the claims. The inclusion of positive valence interventions, to complement the established negative valence ones, seems promising, although the positive valence somatosensory intervention (brushing) seems to have returned ambiguous results. I find the analysis of the data to be moderately lacking and somewhat unclear, both numerically and conceptually, which lowers the resulting impact of the work. With an expanded and refined analysis of the data, I believe this work will be ready to publish, and should be useful for scientists working with mice and other animals in a variety of fields.

Major comments:

Analysis of results and the multi-parameter space of facial expressions:

Plenty of data was collected, but I feel that the numeric analysis should be expanded. These seven parameters define a seven-dimensional space, each experiment a set of points. This form of analysis raises several points which I think are vital to this work: Which directions in this space best represent the positive/negative valence dimension? How are gustatory and somatosensory experiments different in this space? How do individual mice vary in this space? The MGS is discussed, with commentary on how it only has three levels of graduation. How fine-grained are these new measures? These analyses seem both straightforward and important to justify the work. Could a novel face image be measured and placed into this space, and interpreted as coming from a positive or negative experience? Either a random image from these experiments left out from model training, or an image from an experiment from another lab, could be used for this. The latter option would offer an opportunity to address the generalizability of this method, which will be crucial for readers to implement it in their own work. A linear equation could produce a “valence score” which is high for sucrose and habituated petting, and low for formalin and poking. Deviations from this score would themselves be interesting to evaluate. The assertion that the ML algorithms “do not provide an understanding of the processes underpinning facial expressions categorization and interpretation” is belied by that aspect lacking in this work, and I think a more fleshed-out results analysis could help with that.

Machine learning:

Missing from the work is a larger discussion of the trade-offs of this manual technique with, and eventual integration with, automated methods (machine learning). It seems that annotation of a small number of points on fixed images will be done automatically in the near future, improving reproducibility and reducing the cost of these measurements. Additionally, automated and machine-learning-based methods will scale readily to increased frame sampling rate, up to continuous movie analysis, which is infeasible with human-in-the-loop methods. The authors address this topic in a short section near line 73, but given recent rapid advances in the state of the art of machine learning algorithms (see DeepLabCut for body pose, and PainFace for MGS estimation, among others), this section should be expanded. I do believe in the value of analyzing a small number of closely-observed and human-interpretable variables, but the future of experimental image analysis is machine learning and the value of this work should be placed more clearly into the scope of this changing situation.

Minor comments:

Methods:

Most experiments consist of sequences of three interventions. The reasoning for this ordering and choice of combinations should be explained, along with any within-experiment confounding that is expected.

Results:

Results paragraph at line 302, the sentence clauses and numeric claims are oddly ordered. Values should be compared consistently relative to the shared baseline, rather than against each other primarily. The clarity of this analysis would generally be helped by the above point on constructing a 7D (or subset 2D) space of results and showing figures of that, perhaps in a dimensionality-reduced form via UMAP.

Figures:

The baseline should be 1.0 (or whatever value makes sense) with all values presented relative to the baseline, which should be at the middle of the Y-axis. For angles, the reference orientation is arbitrary, and for eye and ear opening the ratio of 1.0 has no special behavioral meaning. This would improve interpretability. The Y scales should be optimized to present data more visibly e.g. 2F can have a Y range of 20 - 50 degrees, zero degrees is an arbitrary value to start the scale at. The box and whiskers should be much more visible over the individual points. The face illustrations are high quality and evocative, nice work.

## Author Response

Answer to reviewers

First, we would like to thank the reviewers for their attention to the manuscript and their constructive feedback. In accordance with their comments, we have made the following changes, highlighted in bold blue in the revised manuscript:

Reviewer 1:

Major issues:

1) Attempting to quantify rodent facial expression by measuring various facial angles is not new. Previous studies (e.g. Finlayson et al., 2016, Plos One; a study not cited by the authors) have approached this question in a very similar manner and even established many, if not most, of the measurements used in this paper. I would suggest the authors carefully consider their claims of novelty and ensure they are accurately positioned in the framework of existing literature.

This paper (Finlayson et al., 2016) is now included in the citations, and our finding during animal brushing and petting discussed in the light of theirs (introduction: l. 65, Methods: l.190, discussion: l. 576), where we refer to the fact that Finlayson et al. also included a positive tactile stimulation (tickling). However, tickling is a stimulus of ambiguous valence and could be considered an extreme somatosensory stimulation, largely different from that produced by gentle touch. Out of the 7 comprehensively defined parameters used in our study, 2 are found in common with the Finlayson study. Those are eye opening (also used in the original MGS paper) and ear position (us)/ear angle (them). Thus, we maintain that most of our facial parameters are new and allow for a better understanding of facial movement associated with different emotional stimuli. The significant results related to ear angle in Finlayson et al. are now mentioned in the manuscript (l. 572) to reinforce the value of this parameter.

2) The method described in this paper would benefit from better justification. The authors suggest that the 7 measurements described in this paper were retained based on extensive measurements. Describing what were the other measurements tested and what were the thresholds for measurement retainment would provide useful information for future facial expression studies and provide support for the validity of the chosen measurements.

Other measurements included the use of different reference points to measure mouth and snout positions, as well as other ear measurements. The parameters retained showed the most significant changes and were the less redundant. This is now explained l. 192.

3) It is not very convincing that all of the 7 measurements are useful and necessary for quantifying expression. The authors state that the measurements are significantly correlated to each other. If that is the case, how many measurements are needed to actually distinguish between the stimuli presented and how many to distinguish between the underlying states? It would be interesting to see dimensionality reduction (for example using PCA) performed on the quantitative measurements. This might also help authors quantify the differences and similarities in the pattern of measurement changes across different stimuli. Currently, these comparisons are rather qualitative and in my opinion insufficient to claim that the method infers emotion states, for which much more evidence would be needed.

Following reviewers’ advice, with used first PCA to reduce dimensionality and used UMAP to derive stimuli clusters (Methods: l. 271). These results are presented in a new result section (3.5) and in figure 8. In addition, according to our first analysis, despite a degree of correlation between the parameters, it appeared that the face areas showing the most significant changes varied with the nature of the stimulus (sensory modality stimulation + valence), arguing in favor of retaining all parameters to obtain a complete picture of facial expressions. This is also mentioned in the discussion, l. 502.

4) Innocuous optogenetic stimulation of WT control mice resulted in what looks like a significant change across the expression measurements. Why?

Innocuous optogenetic stimulation of WT animals produced a single significant difference from baseline values, causing eye closing. This is due to light avoidance, since the light from the optic fiber could be visible to freely moving mice. This is now ackowledged l. 475.

Reviewer 2:

1) Major comments:

Analysis of results and the multi-parameter space of facial expressions:

Plenty of data was collected, but I feel that the numeric analysis should be expanded. These seven parameters define a seven-dimensional space, each experiment a set of points. This form of analysis raises several points which I think are vital to this work: Which directions in this space best represent the positive/negative valence dimension? How are gustatory and somatosensory experiments different in this space? How do individual mice vary in this space? The MGS is discussed, with commentary on how it only has three levels of graduation. How fine-grained are these new measures? These analyses seem both straightforward and important to justify the work. Could a novel face image be measured and placed into this space, and interpreted as coming from a positive or negative experience? Either a random image from these experiments left out from model training, or an image from an experiment from another lab, could be used for this. The latter option would offer an opportunity to address the generalizability of this method, which will be crucial for readers to implement it in their own work. A linear equation could produce a “valence score” which is high for sucrose and habituated petting, and low for formalin and poking. Deviations from this score would themselves be interesting to evaluate. The assertion that the ML algorithms “do not provide an understanding of the processes underpinning facial expressions categorization and interpretation” is belied by that aspect lacking in this work, and I think a more fleshed-out results analysis could help with that.

In response to this comment, first, we used PCA to assess the utility of dimensionality reduction but found that retaining our absolute values would be just as informative. Therefore, we used UMAP to project our set of individual units corresponding to each stimulus in 2-dimensions. The clusterization is overlapping, which we explained by the high variability of our variables in freely-moving animals as well as the overall low number of units (405) and high number of stimuli (1+ baseline) compared to what is often used with UMAP. The clusterization achieved with the current data set still allows to project individual values in space in a way that identifies the sensory modality of the stimulus and in part its valence. Finally, we are of the opinion that emotional valence cannot be described in linear terms, hence the need for the inclusion of more stimuli to produce a more accurate, multi-dimensional projection in space. The results are presented in Fig.8 and l. 439, and further discussed l. 576.

2) Machine learning:

Missing from the work is a larger discussion of the trade-offs of this manual technique with, and eventual integration with, automated methods (machine learning). It seems that annotation of a small number of points on fixed images will be done automatically in the near future, improving reproducibility and reducing the cost of these measurements. Additionally, automated and machine-learning-based methods will scale readily to increased frame sampling rate, up to continuous movie analysis, which is infeasible with human-in-the-loop methods. The authors address this topic in a short section near line 73, but given recent rapid advances in the state of the art of machine learning algorithms (see DeepLabCut for body pose, and PainFace for MGS estimation, among others), this section should be expanded. I do believe in the value of analyzing a small number of closely-observed and human-interpretable variables, but the future of experimental image analysis is machine learning and the value of this work should be placed more clearly into the scope of this changing situation.

We agree that machine learning is the way forward for facial expression analysis among other biobehavioral components. We found that one pitfall of machine learning is its opacity, notably when used on raw data as a tool to extract components of interest. We wanted to use a different approach by identifying and validating our parameters first. From there, machine learning is absolutely implementable and should render data collection and emotion identification much quicker and more accurate. We discuss these points in the discussion, l. 592.

3) Minor comments:

Methods: Most experiments consist of sequences of three interventions. The reasoning for this ordering and choice of combinations should be explained, along with any within-experiment confounding that is expected.

The reasoning for stimuli-sequence ordering is now further explained l. 130.

Results: Results paragraph at line 302, the sentence clauses and numeric claims are oddly ordered. Values should be compared consistently relative to the shared baseline, rather than against each other primarily. The clarity of this analysis would generally be helped by the above point on constructing a 7D (or subset 2D) space of results and showing figures of that, perhaps in a dimensionality-reduced form via UMAP.

A new analysis with UMAP is now visible in Fig. 8. In parallel, some of the results have been re-phrased to improve understandability, l. 319.

Figures: The baseline should be 1.0 (or whatever value makes sense) with all values presented relative to the baseline, which should be at the middle of the Y-axis. For angles, the reference orientation is arbitrary, and for eye and ear opening the ratio of 1.0 has no special behavioral meaning. This would improve interpretability. The Y scales should be optimized to present data more visibly e.g. 2F can have a Y range of 20 - 50 degrees, zero degrees is an arbitrary value to start the scale at. The box and whiskers should be much more visible over the individual points. The face illustrations are high quality and evocative, nice work.

The figures have been modified to improve data visibility. The scales and boxes with whiskers have been optimized where needed. Baseline could not be used as the reference value since some figures presented 2 baselines (for experimental and control groups). Instead, a dash line now represents the average baseline value on Fig. 2, 5, 6 and 7. The ratio of 1 for eye and ear opening corresponds to a completely round unit, which is now explicitly mentioned in the captions of Fig.1.

We hope that the current changes answer all of the reviewers’ concerns, and that the manuscript is suitable for publication.

Sincerely, On behalf of the co-authors.
